# Duck Tembusu Virus Infection Promotes the Expression of Duck Interferon-Induced Protein 35 to Counteract RIG-I Antiviral Signaling in Duck Embryo Fibroblasts

**DOI:** 10.3389/fimmu.2021.711517

**Published:** 2021-07-15

**Authors:** Peng Zhou, Lei Ma, Zaixiao Rao, Yaqian Li, Huijun Zheng, Qigai He, Rui Luo

**Affiliations:** ^1^ State Key Laboratory of Agricultural Microbiology, College of Veterinary Medicine, Huazhong Agricultural University, Wuhan, China; ^2^ Key Laboratory of Preventive Veterinary Medicine in Hubei Province, The Cooperative Innovation Center for Sustainable Pig Production, Wuhan, China

**Keywords:** duck Tembusu virus, quantitative proteomic analysis, interferon-induced protein 35, interferon, RIG-I

## Abstract

Duck Tembusu virus (DTMUV) is an emerging pathogenic flavivirus that has caused a substantial drop in egg production and severe neurological disorders in domestic waterfowl. Several studies have revealed that viral proteins encoded by DTMUV antagonize host IFN-mediated antiviral responses to facilitate virus replication. However, the role of host gene expression regulated by DTMUV in innate immune evasion remains largely unknown. Here, we utilized a stable isotope labeling with amino acids in cell culture (SILAC)-based proteomics analysis of DTMUV-infected duck embryo fibroblasts (DEFs) to comprehensively investigate host proteins involved in DTMUV replication and innate immune response. A total of 250 differentially expressed proteins were identified from 2697 quantified cellular proteins, among which duck interferon-induced protein 35 (duIFI35) was dramatically up-regulated due to DTMUV infection in DEFs. Next, we demonstrated that duIFI35 expression promoted DTMUV replication and impaired Sendai virus-induced IFN-β production. Moreover, duIFI35 was able to impede duck RIG-I (duRIG-I)-induced IFN-β promoter activity, rather than IFN-β transcription mediated by MDA5, MAVS, TBK1, IKKϵ, and IRF7. Importantly, we found that because of the specific interaction with duIFI35, the capacity of duRIG-I to recognize double-stranded RNA was significantly impaired, resulting in the decline of duRIG-I-induced IFN-β production. Taken together, our data revealed that duIFI35 expression stimulated by DTMUV infection disrupted duRIG-I-mediated host antiviral response, elucidating a distinct function of duIFI35 from human IFI35, by which DTMUV escapes host innate immune response, and providing information for the design of antiviral drug.

## Introduction

Tembusu virus (TMUV) is an arbovirus belonging to the Ntaya virus group within the genus *Flavivirus*, family *Flaviviridae* (1), which was first isolated in 1955 from *Culex tritaeniorhynchus* mosquitoes collected in Malaysia (2). In April 2010, a severe outbreak of duck TMUV (DTMUV) infection occurred in the main duck-producing regions of China (3). It caused a substantial drop in egg production and severe neurological disorders in duck population, giving rise to massive economic losses in the duck industry (4–6). Since then, DTMUV has been extensively distributed, leading to pandemic in duck flocks in China and southeastern Asian countries (7, 8). Recent studies reported that DTMUV can infect a wide variety of avian species, including pigeons (4), sparrows (9), geese (10, 11) and chickens (12), and replicate efficiently in a wide range of mammalian cells and mosquito cells (13, 14). Notably, DTMUV can also cause fatal encephalitis and systemic infection in BALB/c and Kunming mice *via* intracerebral inoculation (15, 16). Despite of no report about human disease caused by DTMUV, antibodies against DTMUV were detected in the serum samples of over 70% of duck industry workers while about 50% of oral swab samples were positive for DTMUV RNA test (17). Thus, DTMUV pose a potential threat to public health.

DTMUV contains a single-stranded, positive-sense genomic RNA with an approximate length of 11 kb that encodes a unique large precursor polyprotein (18). The precursor is immediately cleaved by cellular and viral proteases into three structural proteins (capsid [C], precursor membrane [prM], and envelope glycoprotein [E]), and seven nonstructural (NS) proteins (NS1, NS2A, NS2B, NS3, NS4A, NS4B, and NS5) (19, 20). The structural proteins mainly participate in receptor binding, membrane fusion, and virion assembly, while NS proteins are involved in viral genome replication and modulation of host innate immune response (21, 22).

The host innate immunity is the first line defense to combat viral invasion and replication. During flavivirus replication, the viral genome and RNA replication intermediates generated by viral replicase can be recognized by host pattern recognition receptors (PRRs), such as the cytoplasmic RNA helicases: retinoic acid-inducible gene-I (RIG-I) and melanoma differentiation-associated gene 5 (MDA5) (23–25). Upon binding to viral RNA structures, RIG-I and MDA5 become activated and recruit the mitochondrial antiviral signaling protein (MAVS) (26–29), resulting in the activation of inhibitor of κB kinase ϵ (IKKϵ) and TANK binding kinase 1 (TBK1) (30). Subsequently, the transcription factors interferon regulatory factor 3/7 (IRF-3/7) and NF-κB are phosphorylated and translocate to the nucleus, where they bind to the promoter of interferon-beta (IFN-β) and activate its transcription (31). Synthesized IFN-β is secreted, and binds to IFN receptors on the cell surface, triggering hundreds of IFN-stimulated genes (ISGs) expression through Janus kinase/signal transducer and activation of transcription (JAK/STAT) signaling pathway (32, 33).

It has been shown that DTMUV exploits several strategies to subvert innate immune responses. For example, DTMUV NS1 impedes the RIG-I signaling pathway *via* targeting MAVS (34). Moreover, DTMUV NS2B3 protease cleaves duck stimulator of interferon genes (STING), resulting in inhibition of IFN production (22). Interestingly, DTMUV NS2A has also been reported to competitively bind to STING with TBK1, reducing TBK1 phosphorylation and IFN production (21). Besides subversion of host innate immune signaling pathways mediated directly by flavivirus proteins, viral stimulation of the expression of host negative regulatory proteins is an alternative way of interfering with the host innate immune response. For instance, Dengue virus and Zika virus suppress the innate immune response by upregulation of the ubiquitin E3 ligase PDZ and LIM domain protein 2 (PDLIM2) that contributes to ubiquitination of STAT2 and its degradation in cell nucleus (35). However, the role of DTMUV-regulated host gene expression in innate immune evasion remains poorly understood. Therefore, in this study, we conducted a stable isotope labeling with amino acids in cell culture (SILAC)-based proteomics analysis of duck embryo fibroblasts (DEFs) infected with DTMUV and identified 250 host proteins whose expression levels are significantly altered in response to DTMUV. Among the differentially expressed proteins, we found that DTMUV infection strongly induced expression of the duck interferon (IFN)-inducible gene 35 (duIFI35). Importantly, duIFI35 expression could substantially inhibit SeV-induced IFN-β production and greatly facilitate viral replication. Moreover, the interaction of duIFI35 with duck RIG-I (duRIG-I) weakens the interaction between duRIG-I and dsRNA, suppressing the IFN production. Our findings provide new insights on how DTMUV evades host innate immunity by modulation of host gene expression.

## Materials And Methods

### Sample Preparation for Quantitative Proteomic Analysis

For SILAC experiments, DEFs were grown in stable-isotope-labeled Minima Essential Medium (MEM, Gibco, USA) containing either lysine 13C6 and arginine 13C6, 15N4 [“heavy” label (H)] or unlabeled lysine and arginine [“light” label (L)], 10% dialyzed fetal bovine serum (FBS, Gibco, USA) and antibiotics for at least six cell doublings prior to infection. After the full incorporation of heavy amino acids has been verified by MS, heavy-labeled DEFs were infected with DTMUV at an MOI of 0.5, while light-labeled cells were mock-infected to generate reference proteins. At 24 h post-infection (hpi), heavy- and light-labeled DEFs were lysed with 2 × NETN buffer (200 mM NaCl, 100 mM Tris-Cl, 2mM EDTA, 1.0% NP-40, pH 7.2) supplemented with 0.5% Triton X-100 on ice, respectively. After centrifugation, protein concentration of each supernatant was measured by BCA protein assay (Thermo Fisher Scientific, USA). Heavy- and light-labeled proteins in supernatant were mixed at a 1:1 ratio and subsequently precipitated by adding trifluoroacetic acid (TFA) with 15% final concentration (v/v). After two washes with -20℃ acetone, the proteins pellets were dissolved in 100 mM NH_4_HCO_3_ (pH 8.0) for trypsin digestion. To extract the chromatin-bound proteins, the remaining heavy- and light-labeled cell pellets were dissolved in 8 M urea separately. After heavy- and light-labeled chromatin-bound proteins in urea solution were mixed at a 1:1 ratio, the proteins were precipitated by TFA. After washing with acetone, the proteins pellets were also dissolved in NH_4_HCO_3_ for trypsin digestion. Trypsin (Promega, USA) was added into protein solution to a final protease:protein ratio of 1:50 (w/w) for digestion at 37°C for 16 hours. The sample was then fractionated by high pH reverse-phase HPLC using Agilent 300Extend C18 column.

### LC-MS/MS Analysis

For LC–MS/MS analysis, peptides were dissolved in 0.1% formic acid, directly loaded onto a reversed-phase pre-column (Acclaim PepMap 100, Thermo Fisher Scientific, USA). Reversed-phase analytical column (Acclaim PepMap RSLC, Thermo Fisher Scientific, USA) was used for peptide separation as previously reported (36). The resulting peptides were analyzed by Q Exactive™ Plus hybrid quadrupole-Orbitrap mass spectrometer (Thermo Fisher Scientific, USA). Intact peptides were detected in the Orbitrap at a resolution of 70,000 and all the peptides were selected for MS/MS using NCE by setting as 28. Ion fragments were detected in the Orbitrap at a resolution of 17,500. Automatic gain control was employed to prevent overfilling of the ion trap; 5E4 ions were accumulated for generation of MS/MS spectra. For MS scans, the m/z scan range was 350 to 1800.

### Proteome Data Analysis

All MS/MS data were processed using MaxQuant with integrated Andromeda search engine (v. 1.5.3.17). Tandem mass spectra were searched against the UniProt *Galloanserae* database (20,274 sequences) concatenated with reverse decoy database. Trypsin/P was specified as cleavage enzyme allowing up to 2 missing cleavages, 4 modifications per peptide and 5 charges. Mass error was set to 10 ppm for precursor ions and 0.02 Da for fragment ions. Carbamidomethylation on cysteines was considered as fixed modification, while acetylation on protein N-terminal and oxidation on methionine were defined as variable modifications. The cutoff of false discovery rate (FDR) for peptide and protein identification were specified at 1%. Minimum peptide length was set at 7. All the other parameters in MaxQuant were set to default values. The proteins with fold change *≥* 1.5 or *≤* 0.67 in relative abundance (permutation test; *P <* 0.05) were identified as differentially expressed proteins, which were imported into Gene Ontology (GO) and Ingenuity Pathway Analysis (IPA; Qiagen, Germany) for molecular function, biological processes and network analysis.

### Cell, Viruses, and Reagents

DEFs was obtained from American Type Culture Collection (ATCC #CCL-141) and maintained in MEM (Gibco, USA) supplemented with 10% heat-inactivated FBS (Gibco). HEK-293T cells were grown in Dulbecco’s Modified Eagle’s medium (DMEM, Gibco, USA) with 10% FBS. DTMUV strain MC (GenBank Accession Number: KX452096) and Sendai virus (SeV) used in this study were stored in our lab, as previously described (37). Three pairs of siRNA oligonucleotides targeting the duIFI35 gene were synthesized by GenePharma (China) (**Supplemental Table S2**). Poly(I:C) were purchased from Sigma (St Louis, MO, USA). Mouse monoclonal antibodies against Flag, HA, Myc and β-actin were purchased from MBL (Japan). The monoclonal antibody, clone 3F12, against DTMUV E protein was generated previously in our lab. The polyclonal antibody against duIFI35 was prepared *via* the injection of BALB/c mice with His-tagged duIFI35 recombinant protein, which was expressed in *E. coli* Rosetta (DE3) and purified using Ni-NTA affinity chromatography (GE Healthcare, USA).

### Plasmids

DuIFI35 was amplified by standard RT-PCR from total RNA extracted from DEFs and cloned into pCAGGS expression vector with a HA tag at the N-terminus (pCAGGS-HA). Four duIFI35 truncated mutants, including duIFI35 (aa1-164), duIFI35 (aa1-267), duIFI35 (aa123-359), and duIFI35 (aa261-359), were constructed and cloned into pCAGGS-HA. The duck RIG-I, MDA5, MAVS, TBK1, IKKє and IRF7 genes were cloned into the pCAGGS expression vector with a Flag tag at the N-terminus (pCAGGS-Flag). Moreover, duRIG-I truncated mutants (1-200, 201-933, and 736-933 aa) were cloned into the pCAGGS-Flag. The luciferase reporter plasmid IFN-β-Luc has been described previously (38). Sequences of all plasmids were verified by sequencing. The PCR primers used in this study were summarized in **Supplemental Table S1**.

### Indirect Immunofluorescence Assay

DEFs were seeded on coverslips in a 24-well plate and allowed to reach ~80% confluence. Then, the cells were inoculated with DTMUV at an MOI of 0.5. At indicated time post-infection, the infected cells were washed twice with PBS, fixed with 4% paraformaldehyde for 15 min, permeabilized with 0.1% Triton X-100 for 15 min, and blocked with PBS containing 5% bovine serum albumin (BSA) for 1 h. The cells incubated with anti-DTMUV E mAb diluted in 1% BSA for 2 h. After three washes with PBS containing 0.1% Tween 20 (PBST), the cells were incubated with Alexa Fluor 488-conjugated anti-mouse secondary antibody and subsequently treated with 4′,6-diamidino-2-phenylindole (DAPI) for 10 min. Finally, fluorescent signals were captured using a Zeiss LSM 510 Meta confocal microscope (Carl Zeiss, Germany).

### Quantitative Real-Time PCR

Total RNA was prepared from DEFs or tissues from cherry valley ducks using TRIzol reagent (Invitrogen, USA) according to the manufacturer’s instructions. The RNA extracted from each sample was reverse-transcribed to cDNA using Transcriptor First Strand cDNA Synthesis Kit (Roche, Switzerland), and qPCR was performed using FastStart Universal SYBR Green Master Mix (Roche, Switzerland) on ViiA 7 system (Applied Biosystems, USA). The abundances of individual transcripts in each sample were assayed three times and normalized to duck GAPDH (AY436595.1) mRNA level. All primers used for qPCR were listed in **Supplemental Table S1**.

### Luciferase Reporter Assay

DEFs were seeded onto 48-well plates at a density of 5 x 10^4^ cell/well and cultured until the cells reached ~80% confluence. Then, the cells were co-transfected with 50 ng/well of IFN-β-Luc and 50 ng/well of the *Renilla* luciferase-expressing construct pGL4.74 (Promega, USA) as an internal control, together with 300 ng/well of indicated expression plasmids or the pCAGGS vector. 24 h later, the cells were stimulated with SeV. At 16 h post-stimulation, cells were harvested for luciferase assays using Dual-Luciferase Reporter Assay System (Promega, USA) according to the manufacturer’s instructions. The data are represented as the ratio of firefly to *Renilla* luciferase activity.

### Western Blot Analysis

The transfected or DTMUV-infected cells were lysed with lysis buffer [(65 mM Tris–HCl, 4% sodium dodecyl sulfate, 3% DL-dithiothreitol, 40% glycerol, and 1‰ phenylmethyl sulfonyl fluoride] containing protease inhibitor cocktail (Roche, Switzerland). Equal amounts of samples were separated using 12% SDS-PAGE, transferred onto polyvinylidene difluoride membranes (Millipore, USA) and then blocked using 5% nonfat milk in TBST buffer. Specific protein bands were detected using appropriate primary and secondary antibodies, and visualized using an enhanced chemiluminescence (ECL) system (Bio-Rad, USA).

### Co-Immunoprecipitation Assay

HEK-293T cells were transfected with the indicated expression plasmids containing Flag or HA tags. At 30 h post-transfection, the cells were lysed in RIPA buffer [50 mM Tris-HCl (pH 7.4), 150 mM NaCl, 1% Triton X-100, 1% sodium deoxycholate, 0.1% SDS and 2 mM EDTA] with protease inhibitor cocktail (Roche) and incubated on ice for 30 min. For each sample, 0.4 ml of cell lysate was incubated with 10 μg of anti-Flag mAb at 4°C for 8 h and with 1 mg of protein A/G plus-agarose (Santa Cruz Biotechnology, USA) for 2 h at room temperature. The Sepharose beads were washed 5 times with cold RIPA buffer. The precipitates were fractionated by SDS-PAGE and subsequently analyzed using standard immunoblot procedures.

### Poly(I·C) Pulldown Assay

DEFs cultured in 60-mm plates were transfected with the indicated expression plasmids or empty vector for 28 h. Then, the cells were lysed in lysis buffer (50 mM Tris [pH 7.4], 150 mM NaCl, 1% sodium deoxycholate, 2 mM EDTA, 1% NP-40, 0.1% SDS) containing protease inhibitor cocktail (Roche). After centrifugation, the supernatants were incubated with a prepared suspension of poly(I·C)-coated agarose beads for 4 h at 4°C. The beads were washed three times with lysis buffer by resuspension and centrifugation, and subsequently subjected to standard Western blotting procedures using anti-Flag mAb (MBL, Japan) as the primary antibody.

### Statistical Analysis

All statistical analyses were performed using GraphPad Prism software (GraphPad Software, Inc.). An unpaired Student’s *t*-test was employed to determine the *P*-value. *P*-values *<* 0.05 were considered statistically significant and *P*-values *<* 0.01 were highly significant.

## Results

### Identification of Differentially Expressed Proteins in DTMUV-Infected DEFs

**Figure 1:**
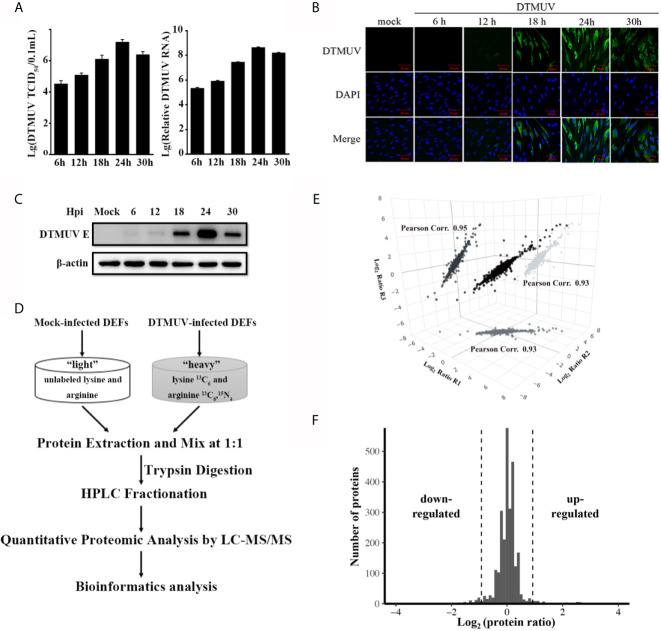
Quantitative proteomic analysis of DTMUV-infected DEFs. **(A)** DEFs were inoculated with DTMUV (MOI=0.5), followed by collection at the indicated time points. Virus titers in the cell culture was determined by TCID_50_ assay and intracellular RNA was extracted for the measurement of viral genome RNA by RT-qPCR. Data represent the mean and SD of three independent experiments. **(B, C)** Dynamics of DTMUV replication in DEFs was analyzed by immunofluorescence staining **(B)** and Western blotting **(C)**. **(D)** SILAC-based quantitative proteomic workflow for DTMUV-infected DEFs. **(E)** The correlation of protein ratios from three biological replicates. Pearson correlation efficiencies was calculated based on log2 protein ratio from two independent biological replicates. **(F)** The Gaussian distribution of protein ratios.

To find host proteins that can modulate DTMUV-induced innate immune response and influence DTMUV replication, we employed SILAC-based quantitative proteomics to identify differentially expressed host proteins upon DTMUV infection. Before proteomic analysis, we monitor the kinetics of DTMUV replication *via* TCID_50_ assay and RT-qPCR to determine the time points of high viral replication activity. As shown in **Figure 1A**, after infection, intracellular genomic RNA level of DTMUV and viral titer increased until 24 h post-infection (hpi), and then dropped at 30 hpi in DEFs. The specific immunofluorescence corresponding to viral envelope protein was readily detected in almost all DEFs infected with DTMUV at 24 hpi (**Figure 1B**), whereas, at this time, there was no significant difference of the morphology between the mock and DTMUV infected cells (data not shown). Moreover, according to Western blot, the expression of viral envelope protein in the DTMUV-infected DEFs also peaked at 24 hpi (**Figure 1C**). As a result, 24 hpi was chosen for our quantitative proteomics analysis. Proteins from DTMUV- and mock-infected DEFs were extracted and subsequently digested with trypsin, followed by peptide fractionation and analysis of the resulting peptides through liquid chromatography coupled with MS/MS (LC-MS/MS) (**Figure 1D**). Three independent biological replicates were performed, and Pearson correlation efficiencies between replicates were calculated based on the protein ratios (**Figure 1E**). The Gaussian distribution of protein ratios was analyzed, and proteins with ratios deviating from the mean of the normally distributed data by 1.96 standard deviations (SDs) were considered differentially regulated (**Figure 1F**). Consequently, we identified a total of 5627 proteins, among which 2697 proteins were quantified with a false discovery rate of 1%. All quantified proteins with their ratios and SD values were listed in **Supplemental Table S3**. Among the quantified proteins, 112 proteins were up-regulated (fold change >1.5, *P <* 0.05) and 138 proteins were down-regulated (fold change *<* 0.67, *P <* 0.05) upon DTMUV infection at 24 hpi (**Supplemental Table S4**).

### Validation of Differentially Expressed Proteins by RT-qPCR and Western Blot

**Figure 2:**
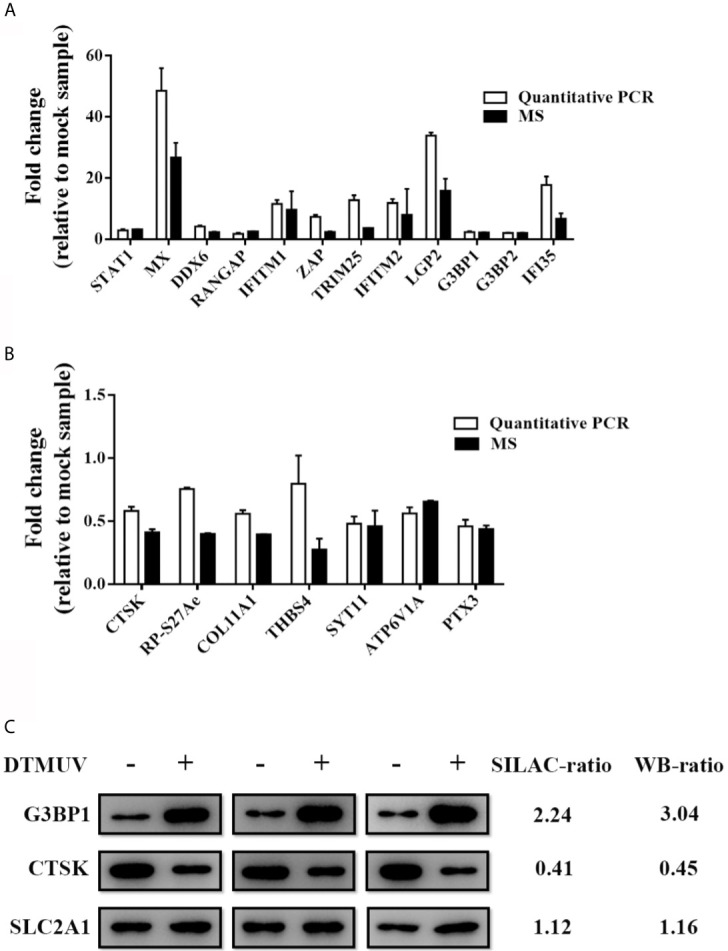
Validation of MS results by RT-qPCR and Western blotting. DEFs were inoculated with DTMUV (MOI=0.5) and were harvested at 24 hpi. **(A, B)** Intracellular mRNAs were extracted for RT-qPCR for measuring the mRNA of up-regulated **(A)** and down-regulated **(B)** proteins. Data represent the mean and SD of three independent experiments. **(C)** Western blotting analysis of host proteins in DTMUV-infected DEFs. SILAC- and immunoblotting-ratios (infection/control) were shown on the right side. Western blotting results are presented from three independent assays.

To verify the LC-MS/MS data, representative host proteins were subjected to RT-qPCR and western blot analysis. RT-qPCR confirmed the upregulation of duck STAT1, MX, DDX6, RANGAP, IFITM1, ZAP, TRIM25, IFITM2, LGP2, G3BP1, G3BP2 and IFI35 (**Figure 2A**) and the downregulation of duck CTSK, RP-S27Ae, COL11A1, THBS4, SYT11, ATP6V1A and PTX3 as a result of DTMUV infection in DEFs (**Figure 2B**). Western blotting results validated the elevated G3BP1 expression, the decreased CTSK expression, and the unchanged SLC2A1 expression between DTMUV-infected and mock-infected DEFs (**Figure 2C**). These data confirmed the SILAC combined with LC-MS/MS analysis.

### Bioinformatic Analysis of Differentially Regulated Proteins

**Figure 3:**
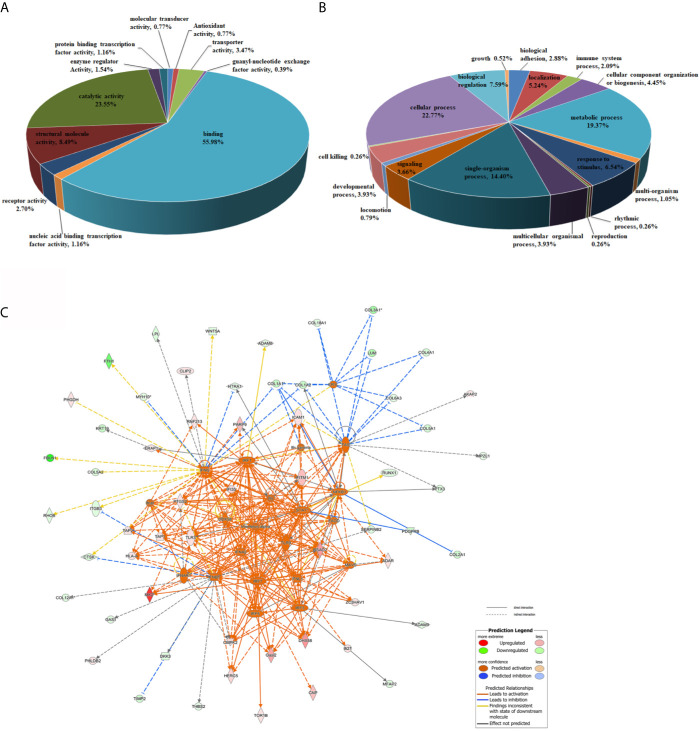
Bioinformatic analysis of differentially regulated proteins. **(A, B)** GO analysis of the differentially regulated proteins in DTMUV-infected DEFs. Proteins were classified according to their Molecular Function **(A)** and Biological processes **(B)**. **(C)** Network analysis of proteins significantly changed in DTMUV-infected DEFs by IPA. Up-regulated and down-regulated proteins are shown in red and green, respectively. The color depth indicates the magnitude of the change in protein expression, and the shapes represent the molecular class. Lines with arrows connecting between the molecules indicate molecular relationships. Solid lines represent direct interactions, and dashed lines represent indirect interactions.

To comprehensively gain biological insight into the proteome data, the differentially regulated proteins in DTMUV-infected DEFs were analyzed for biological functions using GO analysis. As shown in **Figure 3A**, the molecular function categories for regulated proteins mainly included binding proteins (55.98%), catalytic proteins (23.55%), and structural molecule proteins (8.49%). For biological process annotation, the identified proteins were mainly involved in cellular process (22.77%), metabolic process (19.37%), single-organism process (14.4%), biological regulation (7.59%), and response to stimulus (6.54%) (**Figure 3B**). Of note, we observed that several duck IFN-stimulated genes (ISGs), including IFI35, Mx, ISG15, ZC3HAV1, LGP2, IFITM1, IFITM2, and Viperin, were dramatically upregulated upon DTMUV infection (**Supplemental Table S4**). Surprisingly, we also found that duck STAT1, a key regulator of ISGs, was increased 3.25-fold during DTMUV infection (**Supplemental Table S4**), indicating that DTMUV probably induce ISGs expression *via* the activation of canonical JAK-STAT pathway. To comprehensively understand how DTMUV regulates the innate immune response, network analysis was performed through the use of IPA. As shown in **Figure 3C**, the represented networks further revealed that DTMUV infection probably activate production of type I IFNs *via* duck TLR3 and DDX58 (also called RIG-I), triggering the expression of various duck ISGs, such as IFI35, IFITM1, ZC3HAV1 and DHX58 (also named LGP2). Notably, according to our proteome data, the expression level of duIFI35 increased 6.72-fold upon DTMUV infection. Human IFI35 was reported recently to negatively regulate the RIG-I antiviral signaling pathway and facilitate vesicular stomatitis virus replication (39), which is different to the majority of ISGs in antagonizing virus infections. Therefore, we decided to investigate the effect of duIFI35 on DTMUV replication, which, to the best of our knowledge, remained largely unknown.

### IFI35 Promotes DTMUV Infection

**Figure 4:**
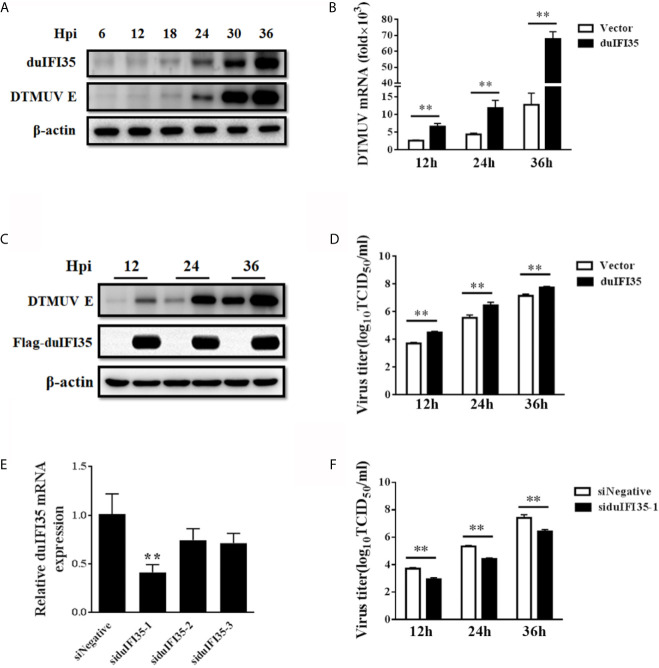
DuIFI35 promotes DTMUV infection. **(A)** DEFs were infected with DTMUV (MOI=0.1) or mock-treated. Cells were harvested at the indicated time points to determine duIFI35 protein expression by western blotting. **(B–D)** DEFs were transfected with Flag-duIFI35 or an empty vector, followed by DTMUV (MOI = 0.1) inoculation at 24 h post-transfection. At the indicated time points, viral RNA, E protein expression, and viral titer were determined by RT-qPCR **(B)**, Western blot **(C)** and TCID_50_ **(D)**, respectively. **(E)** DEFs were transfected with siRNA targeting duIFI35 or control siRNA (siNegative). At 30 h post-transfection, intracellular mRNAs were extracted to analyze the abundance of duIFI35 mRNA by RT-qPCR. **(F)** DEFs were transfected with the siduIFI35-1 or siNegative, followed by DTMUV (MOI = 0.1) inoculation at 24 h post-transfection. Tissue culture was harvested at the indicated time points and the virus titer was measured by TCID_50_. Data represent the mean and SD of three independent experiments. **P < 0.01 (unpaired Student’s t-test).

To further investigate the dynamic change of duIFI35 during DTMUV, Western blotting was employed to detect the expression of duIFI35 in DEFs at different times after DTMUV infection using our home-made anti-duIFI35 polyclonal antibody. In agreement with the results of the proteome data, the amount of the duIFI35 protein was gradually increased during DTMUV infection and peaked at 36 hpi (**Figure 4A**). To evaluate the effect of duIFI35 expression on DTMUV replication, DEFs overexpressing duIFI35 were infected with DTMUV at an MOI of 0.1. Cell cultures were collected at the indicated time, then the viral RNA, E protein expression, and viral titer were determined respectively by RT-qPCR, Western blot and TCID_50_ assays. As illustrated in **Figures 4B–D**, the amount of viral RNA, E protein expression and the viral titer in duIFI35-expressed cells were dramatically elevated compared with the control cells, suggesting that overexpression of duIFI35 in DEFs facilitates DTMUV replication. Moreover, three siRNAs targeting different regions of duIFI35 mRNA were synthesized to further study the effect of endogenous duIFI35 on DTMUV production. Among them, siduIFI35-1 significantly degraded the endogenous mRNA of duIFI35 compared to siNegative control (**Figure 4E**), therefore siduIFI35-1 was chosen in the following knockdown experiments. We found that knockdown of duIFI35 greatly lowered DTMUV titers (**Figure 4F**), suggesting that duIFI35-1 may promote DTMUV infection.

### duIFI35 Suppresses IFN-β Production by Targeting RIG-I Signaling

**Figure 5:**
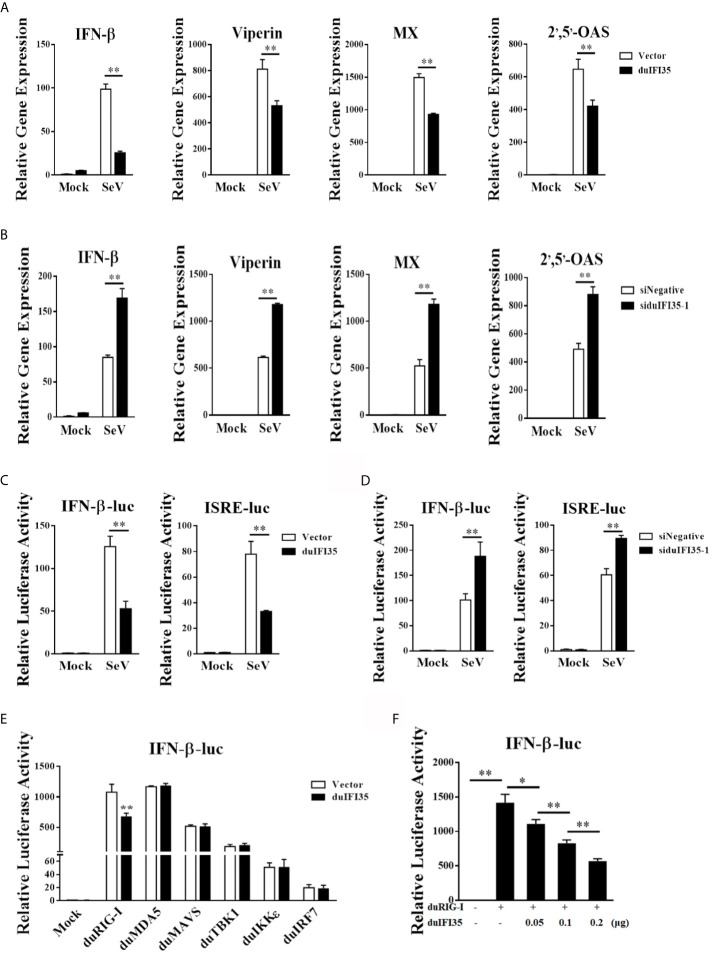
DuIFI35 suppresses IFN-β production by targeting RIG-I signaling. **(A, B)** DEFs were transfected with Flag-duIFI35 **(A)** or siduIFI35-1 **(B)**, followed by SeV infection at 24 h post-transfection. The expression of IFN-β, Viperin, MX and 2^’^,5^’^-OAS mRNA was measured by qRT-PCR and normalized to GAPDH expression. **(C, D)** DEFs were transfected with pRL-TK, IFN-β-Luc or ISRE-Luc together with Flag-duIFI35 **(C)** or siduIFI35-1 **(D)**. At 24 h post-transfection, cells were stimulated with SeV. Luciferase assays were performed 16 h after SeV stimulation. **(E)** DEFs were transfected with Flag-duIFI35, IFN-β-Luc and pRL-TK, along with the plasmids expressing key molecules in RLR signaling. Luciferase assays were performed at 30 h after transfection. **(F)** DEFs were transiently transfected with duRIG-I, IFN-β-Luc and pRL-TK, together with increasing amounts of Flag-duIFI35. Luciferase assays were performed at 30 h after transfection. Data represent the mean and SD of three independent experiments. *P < 0.05; **P < 0.01 (unpaired Student’s t-test).

To investigate whether duIFI35 facilitated DTMUV replication by regulating the host innate immune response, we began by examining the mRNA production of IFN-β, viperin, MX, and 2’,5’-OAS stimulated by SeV in DEFs expressing duIFI35 by RT-qPCR. As illustrated in **Figure 5A**, SeV-induced the mRNA expression of IFN-β, viperin, MX, and 2’,5’-OAS were dramatically decreased in the duIFI35-expressing DEFs when compared to those in empty vector-transfected cells. Additionally, the siRNA-mediated knockdown of duIFI35 in DEFs greatly enhanced mRNA expression levels of IFN-β, viperin, MX, and 2’,5’-OAS induced by SeV (**Figure 5B**). To further investigate whether duIFI35 expression regulates the IFN-β and ISGs production, DEFs were co-transfected with duIFI35 expression plasmid and a luciferase reporter harboring the duck IFN-β or ISRE promoter, followed by SeV infection. Our results showed that duIFI35 expression significantly weakened the SeV-induced activation of IFN-β or ISRE promoter (**Figure 5C**). Moreover, the induced activity of IFN-β or ISRE promoter by SeV infection was prominently enhanced following knockdown of duIFI35 expression in DEFs (**Figure 5D**). These results suggested that duIFI35 expression inhibited dsRNA-induced IFN-β production and host antiviral immune response. To identify the molecular target of duIFI35 in the duIFN-β induction signaling pathway, the plasmids expressing key molecules in RLR signaling, including duRIG-I, duMDA5, duMAVS, duTBK1, duIKKϵ, and duIRF7, were co-transfected with the duIFI35 expression plasmid and a luciferase reporter containing the duIFN-β promoter. The luciferase assays showed that duIFI35 only attenuated the activation of duIFN-β promoter triggered by duRIG-I, while it had no effect on the duIFN-β promoter activity induced by duMDA5, duMAVS, duTBK1, duIKKϵ, and duIRF7 (**Figure 5E**). We also observed that duIFI35 inhibited duRIG-I-induced IFN-β promoter activation in a dose-dependent manner (**Figure 5F**). Collectively, these results indicated that duIFI35 might target duRIG-I to disrupt the duIFN-β production.

### duIFI35 Protein Interacts With duRIG-I

**Figure 6:**
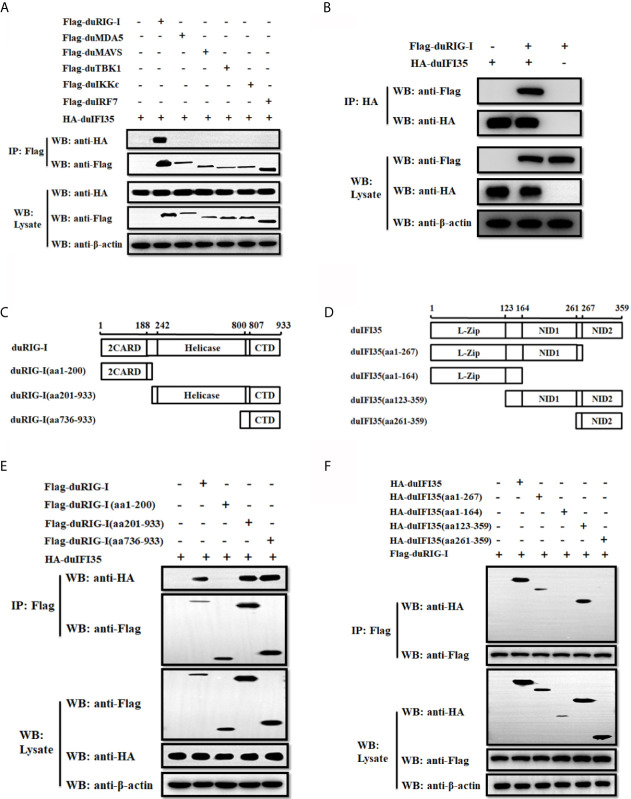
DuIFI35 protein interacts with duRIG-I. **(A)** HEK-293T cells were transfected with HA-tagged duIFI35 plasmid, along with Flag-tagged duRIG-I, duMDA5, duMAVS, duTBK1, duIKKϵ, or duIRF7 plasmid. Cell lysates were immunoprecipitated with anti-Flag antibody. Immunoprecipitation (IP) and immunoblot analyses were performed with anti-Flag, anti-HA, or anti-β-actin antibodies. **(B)** HEK-293T cells were co-transfected with Flag-duRIG-I and HA-duIFI35. Cell lysates were immunoprecipitated with anti-HA antibody, and the IP complexes were detected by Western blotting using anti-Flag and anti-HA antibodies. **(C, D)** Schematic diagram of the full-length duRIG-I **(C)** and duIFI35 **(D)**, their deletion mutants. **(E)** HEK-293T cells were transfected with full-length HA-tagged duIFI35 along with full-length Flag-tagged duRIG-I or its truncations. **(F)** HEK-293T cells were transfected with full-length Flag-tagged duRIG-I together with full-length HA-tagged duIFI35 or its truncations. Cell lysates were immunoprecipitated with anti-Flag antibody, and the IP complexes and the plasmids expression were detected by Western blotting using anti-Flag and anti-HA antibodies.

To explore whether duIFI35 interacts with the components of the RLR signaling pathway, HEK-293T cells were cotransfected with the plasmids encoding HA-tagged duIFI35 and Flag-tagged duRIG-I, duMDA5, duMAVS, duTBK1, duIKKϵ, and duIRF7, and the cell lysates were subjected to co-immunoprecipitation assays using anti-FLAG antibody. As illustrated in **Figure 6A**, duIFI35 interact specifically with duRIG-I, rather than other signaling components. The interaction between duIFI35 and duRIG-I were also confirmed by a reverse co-immunoprecipitation assay with an anti-HA antibody (**Figure 6B**). DuRIG-I contains two N-terminal caspase-recruitment domains (2CARD), a central DExD/H-box helicase domain (Hel), and a C-terminal domain (CTD) (40). To determine which domain of duRIG-I was responsible for binding to duIFI35, a series of duRIG-I mutants lacking the regions encoding one or two domains were constructed (**Figure 6C**). HEK-293T cells were transiently co-transfected with the plasmids expressing Flag-tagged duRIG-I mutants and HA-tagged duIFI35, followed by co-immunoprecipitation analyses with anti-Flag antibody. We observed that duIFI35 was co-immunoprecipitated with the Hel and CTD, or CTD of duRIG-I, but not with the CARD domains (**Figure 6E**), indicating that the CTD of duRIG-I is essential for binding to duIFI35. To identify the domains of duIFI35 binding to duRIG-I, several duIFI35-truncated mutants lacking different domains, such as N-terminal L-Zip domains, NID1 domain, and C-terminal NID2 domain, were constructed (**Figure 6D**). Anti-FLAG co-immunoprecipitation assays were performed by co-expressing Flag-tagged duRIG-I and HA-tagged duIFI35 mutants in HEK-293T cells. We found that the NID1 domain located on the middle of duIFI35 was crucial for its interaction with duRIG-I (**Figure 6F**). Collectively, our results suggested that the interaction between duIFI35 and duRIG-I was mediated by the NID1 in duIFI35 and the CTD in duRIG-I.

### duIFI35 Attenuates the Interaction of dsRNA With duRIG-I

**Figure 7:**
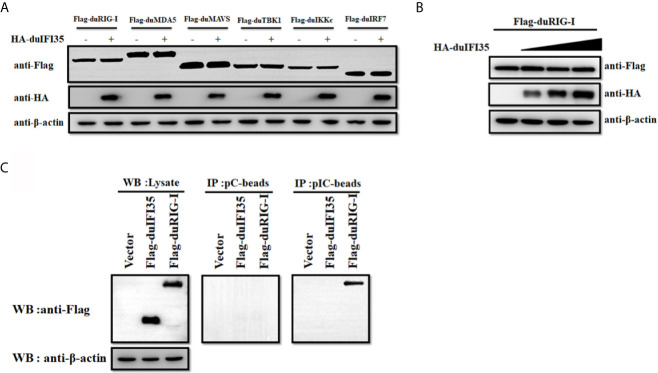
DuIFI35 is not an RNA binding protein. **(A)** DEFs were transfected with HA-duIFI35 along with the plasmids expressing key molecules in RLR signaling. The expression levels of these components in RLR signaling were detected by Western blotting using anti-Flag antibody. **(B)** DEFs were transfected with Flag-duRIG-I along with increasing amounts of HA-duIFI35. DuRIG-I expression were analyzed by immunoblotting with anti-Flag antibody. **(C)** DEFs were transfected with Flag-duIFI35, Flag-duRIG-I, or empty vector, separately. DEFs were lysed at 24 h post-transfection, and the resulting supernatants were incubated with poly(C)- or poly(I·C)-coated agarose beads. After incubation for 4 h at 4°C, the beads were washed three times with lysis buffer and were subjected to Western blot analysis using anti-Flag antibody.

**Figure 8:**
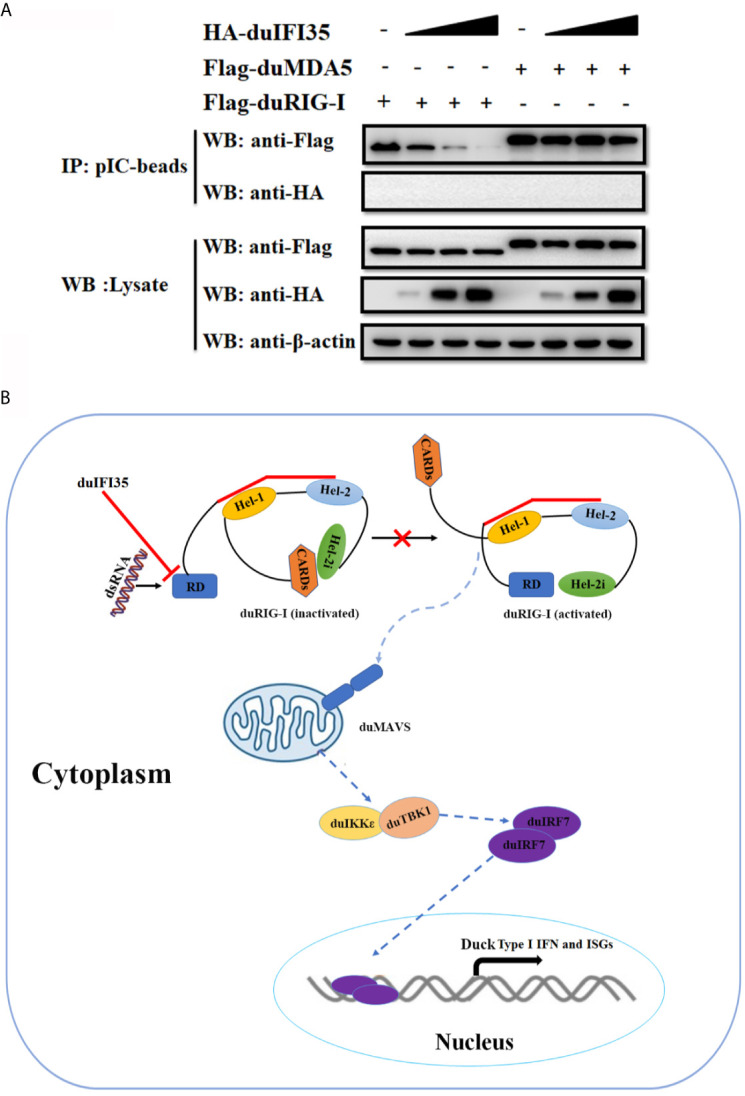
DuIFI35 attenuates the interaction of dsRNA with duRIG-I. **(A)** DEFs were individually transfected with Flag-duRIG-I, Flag-duMDA5, or increasing amounts of HA-duIFI35. At 24 h post-transfection, lysates from the DEFs expressing duIFI35 were mixed with an equal volume of lysates of DEFs transfected with Flag-duRIG-I or Flag-duMDA5, followed by incubation with poly(I·C)-coated agarose beads. The beads were washed four times with lysis buffer and analyzed by Western blotting using anti-Flag antibody. **(B)** A schematic diagram to illustrate how duIFI35 negatively regulates duRIG-I antiviral signaling.

Since previous research showed that human IFI35 induce proteasomal degradation of human RIG-I, we examined whether duIFI35 promotes degradation of duRIG-I (39). However, to our surprise, when plasmids encoding duIFI35 and the signaling molecules of RLR pathway were transfected into DEFs, duIFI35 had no impact on expression of duRIG-I and other signaling molecules as well, including duMDA5, duMAVS, duTBK1, duIKKє, and duIRF7 (**Figure 7A**). Moreover, although the increased amount of duIFI35 expression plasmid was adopted, we still cannot detect the degradation of duRIG-I (**Figure 7B**). Based on the protein function prediction of duIFI35 in UniProt (UniProtKB: A0A6J3E669), duIFI35 had a nucleotide-binding domain. Thus, we investigated whether duIFI35 attenuated duRIG-I-mediated duIFN-β production by competitively binding to dsRNA with duRIG-I. As illustrated in **Figure 7C**, duRIG-I was found to be combined with poly(I·C)-coated, rather than poly(C)-coated, agarose beads, verifying the dsRNA binding to duRIG-I. Unexpectedly, neither poly(I·C)- nor poly(C)-coated agarose beads was interacted with duIFI35 (**Figure 7C**), indicating that duIFI35 is not an RNA-binding protein. Therefore, competition of duIFI35 with duRIG-I for binding to dsRNA is not true for duIFI35-induced disruption of duRIG-I-mediated duIFN-β production. Next, we wonder whether duIFI35 is capable of interfering with the process of dsRNA recognition by duRIG-I. A pulldown experiment with poly(I·C) beads was performed in DEFs. DuMDA5, which is known to bind with poly(I·C), was applied as a control. As shown in **Figure 8A**, duRIG-I binding to poly(I·C) was significantly blocked by duIFI35 in a dose-dependent manner; in contrast, duMDA5 interacting with poly(I·C) was not attenuated by duIFI35, even with a high expression level of duIFI35. Altogether, our results indicated that duIFI35 expression stimulated by DTMUV infection can impair the recognition of dsRNA by duRIG-I, result in the inhibition of duIFN-β production (**Figure 8B**).

## Discussion

As an emerging avian flavivirus, DTMUV has become one of the most deleterious infectious agents, causing considerable economic losses in the poultry industry. Like many other flaviviruses, DTMUV RNA structures produced during viral replication can be detected by RLRs and TLRs, leading to the production of type I IFN and a wide range of ISGs; however, DTMUV have developed several ways to subvert the host innate immune response, thus enabling viral replication in a more effective manner. Previous studies have reported that DTMUV NS1 protein inhibits the RLR signaling pathway by disrupting the interaction between RIG-I/MDA5 and MAVS (34), while STING-induced type I IFN signaling was interrupted by DTMUV NS2A and NS2B3 proteins (21, 22). Despite these DTMUV proteins are implicated in the disruption of the host innate immune response, the roles of host gene expression modulated by DTMUV in innate immune evasion remain largely unknown. To better understand the mechanisms underlying DTMUV innate immune evasion and pathogenesis, here we performed a quantitative proteomic analysis of DTMUV-infected cells to identify critical proteins and intracellular pathways regulated by DTMUV. Our proteomic analysis showed that 112 cellular proteins were upregulated (fold change *>* 1.5, *P <* 0.05) and 138 cellular proteins were downregulated (fold change *<* 0.67, *P <* 0.05) in DTMUV-infected DEFs at 24 hpi (**Supplemental Table S4**). By analyzing the protein network of these differentially expressed proteins with IPA, we found that DTMUV infection might activate RLR and TLR3 signaling pathways leading to the production of type I IFN (**Figure 3C**). A previous report showed that DTMUV infection stimulates type I and III IFNs production *via* MDA5 and TLR3 signaling cascades through shRNA-based knockdown of MDA5 and TLR3 in the 293T cells (41). It is noteworthy that a number of ISGs, such as duck IFI35, Mx, ISG15, ZC3HAV1, LGP2, IFITM1, IFITM2, and viperin, were upregulated according to our proteomics dataset (**Supplemental Table S4** and **Figure 3C**). Meanwhile, duck STAT1 expression was also increased during DTMUV infection (**Supplemental Table S4** and **Figure 3C**), suggesting that DTMUV activates JAK-STAT signaling and induces the production of numerous ISGs. Among these ISGs, the antiviral activity of duck Mx, IFITM1, IFITM2, and viperin against DTMUV has been described in previous studies (42, 43), while the biological functions of avian ISG15, ZC3HAV1, and LGP2 have been extensively decoded in the past decade (44–47). However, the roles of avian IFI35 in antagonizing viral replication remain unexplored. In this study, we found that DTMUV induced duIFI35 expression at different post-infection times (**Figures 4A, B**). Overexpression of duIFI35 dramatically promoted DTMUV replication, whereas knockdown of duIFI35 decreased the viral titers (**Figures 4C** **–F**), indicating that DTMUV facilitate the replication by inducing duIFI35 expression.

It is known that human IFI35 can attenuate SeV-mediated IFN-β response and support vesicular stomatitis virus infection (39). Here, we investigate whether duIFI35 negatively regulates the production of duck IFN-β and the innate antiviral response to efficiently promote DTMUV replication. As expected, duIFI35 expression significantly impairs the expression of IFN-β and ISGs, such as Viperin, MX, and 2’,5’-OAS, stimulated by SeV (**Figures 5A–D**). The recognition of human IFI35 with human RIG-I promotes IFI35 degradation *via* the ubiquitin-proteasome pathway, damping RIG-I-mediated antiviral immune response (39). Our results presented here also showed that duIFI35 interacts with duRIG-I and then inhibits duRIG-I-induced IFN-β production (**Figures 5E, F** and **6**). Moreover, we found that NID1 in duIFI35 and the CTD in duRIG-I are responsible for duIFI35/duRIG-I interaction. However, we did not observe the degradation of duRIG-I mediated by duIFI35 (**Figures 7A, B**), indicating that human and duck IFI35s adopt different strategies to modulate host RIG-I signaling. DuIFI35 shares only 39% amino acid sequence similarity with human IFI35, and duRIG-I possesses 54% similarity with human RIG-I, which may contribute to this difference. In addition, some host factors in ubiquitin-proteasome system (UPS) are possibly diverse between human and duck cells. Potentially, the missing or divergence of certain factors in the duck UPS might eventually affect duRIG-I ubiquitination and degradation by duIFI35.

During flavivirus infection, dsRNA replication intermediates are readily captured by the CTD of RIG-I in the host cells, which subsequently induces a conformational change on RIG-I and releases its N-terminal 2CARD (48, 49). Upon RIG-I activation, 2CARD interacts with the CARD domain of MAVS on the mitochondria, initiating the production of type I IFN and ISGs (50, 51). Human protein activator of PKR (PACT), identified as a dsRNA binding protein, associates with the CTD of human RIG-I and stimulates RIG-I’s ATPase activity, maintaining it in an active state (52). In contrast to PACT, TAR-RNA-binding protein (TRBP) is also a dsRNA binding protein that binds to the RIG-I’s CTD; however, it blocks RIG-I-induced IFN-β expression requiring its dsRNA-binding activity (53). In our study, duIFI35 cannot be combined with either poly(I·C) or poly(C), suggesting it is not an RNA-binding protein (**Figure 7C**). Our results clearly demonstrate that duIFI35 interacting with the CTD of duRIG-I significantly blocks the dsRNA binding with duRIG-I, whereas duIFI35 expression did not attenuate the duck MDA5 binding to dsRNA (**Figure 8A**). Recently, porcine deltacoronavirus NS6 was reported to interact with both RIG-I and MDA5 and negatively modulate RLR signaling pathway by impeding dsRNA recognition by RIG-I and MDA5 (54). To date, few studies have been conducted to clarify how virus evades RIG-I recognition of its dsRNA during genome replication by manipulating the expression of host proteins, especially in avian. To the best of our knowledge, this is the first report of an avian protein that attenuates avian RIG-I signaling by interacting with avian RIG-I and blocking its binding with dsRNA.

In conclusion, we report that DTMUV-induced duIFI35 expression interrupts duRIG-I-mediated duIFN-β and ISGs production by interacting with duRIG-I to attenuate its recognition of dsRNA. Moreover, we identify 250 differentially expressed host proteins upon DTMUV infection by SILAC-based quantitative proteomics. Our results support a continued in-depth investigation on the mechanism of these DTMUV-manipulated host proteins involving innate immune response and viral replication, which facilitates the discovery of effective therapeutic agents and the development of vaccines.

## Data Availability Statement

The datasets presented in this study can be found in online repositories. The names of the repository/repositories and accession number(s) can be found in the article/**Supplementary Material**.

## Author Contributions

RL and QH conceived and supervised the research. PZ, LM, ZR, YL, and HZ performed research. PZ, LM, YL, HZ, and RL analyzed data. RL and PZ wrote the manuscript. All authors contributed to the article and approved the submitted version.

## Funding

This work was supported by grants from National Natural Science Foundation of China (31772737) and the Fundamental Research Funds for the Central Universities (Grant No. 2662019PY078).

## Conflict of Interest

The authors declare that the research was conducted in the absence of any commercial or financial relationships that could be construed as a potential conflict of interest.
