# Supplementary material for: Duck Tembusu Virus Infection Promotes the Expression of Duck Interferon-Induced Protein 35 to Counteract RIG-I Antiviral Signaling in Duck Embryo Fibroblasts
Source: Front Immunol. 2021 Jul 15;12:711517. doi: 10.3389/fimmu.2021.711517 (PMC8320746; doi:10.3389/fimmu.2021.711517)
Supplement: Supplementary file 1 [file Table_1.docx]

**Table S1**

Sequences of the primers used for real-time PCR and construction of plasmids

| Primer | Sequence (5’-3’) |
| --- | --- |
| duIFI35-CDS-F | TATGAATTCATGGACTCGGAGGAGAGCTCC |
| duIFI35-CDS-R | TATCTCGAGCTACTCGCCGAACACAGCCAC |
| duIFI35(aa1-267)-R | TATCTCGAGTTAGAACTCCCTGCTCTCCACCTCGC |
| duIFI35(aa1-164)-R | TATCTCGAGTTATGAGCCGCCCTGCAGAGG |
| duIFI35(aa123-359)-F | TATGAATTCATGCCGATGATGATGTTGCCTGCC |
| duIFI35(aa261-359)-F | TATGAATTCATGGTGGAGAGCAGGGAGTTCCTG |
| qduSTAT1-F | AAGGGAAACGGCTACATCAAG |
| qduSTAT1-R | CATGGGCAGCAGATTTTCTG |
| qduMX-F | TGTCCATGAAACTGCACGAA |
| qduMX-R | TGCTCTCAGGCTGTCAATGA |
| qduDDX6-F | TGAGCGTCAGAAGGTTCATTG |
| qduDDX6-R | ACCCGTTGAGAGGAGTTGCA |
| qduRANGAP-F | AGACGGTGTGCGTGGCTTT |
| qduRANGAP-R | ATGCCCATGCCACAGTTATTG |
| qduIFITM1-F | CCCTGCTGCTCAACATCTTC |
| qduIFITM1-R | GCTGATGGTGGAGCAGGTT |
| qduIFITM2-F | TCTCTGGTCCCTCTTCAACGT |
| qduIFITM2-R | GGCCTTGATGGAGAAGATGAG |
| qduZAP-F | GCTCCTCTTCATTGCTTCGA |
| qduZAP-R | CCACTGGCCTTGGTCATTCT |
| qduTRIM25-F | GAGAGGAGCTTTTGCAGTATGC |
| qduTRIM25-R | GACAGACATCTTGGTGTATCTCTCAGA |
| qduLGP2-F | CGTTTACTTCTACTGCGTCAACTG |
| qduLGP2-R | GTTGATGTTCACGTGGTGCAT |
| qduG3BP1-F | AAACTTCAAGGACTGCCACACA |
| qduG3BP1-R | AGCTCTCCCATCACCTGGACTA |
| qduG3BP2-F | TTCAGTGAGTGCCACACCAA |
| qduG3BP2-R | CGACAGCTCCCCCATAACTT |
| qduIFI35-F | GGATACAACAGGTGAAGAAC |
| qduIFI35-R | ACTGGAGGGATTGGATTAG |
| qduCTSK-F | GGAGGCTGATTTGGGAGAAA |
| qduCTSK-R | TCATGGCCAGCTCAAAGGTA |
| qduIFNβ-F | ACATCCTTTTGGACACCGACAA |
| qduIFNβ-R | TTGGACTGCTGAGGATGTTGA |
| qduRP-S27Ae-F | GCTCAAGTACTACAAGGTGGATGAG |
| qduRP-S27Ae-R | CATGAAGACTCCTGCTCCACACT |
| qdu2’,5’-OAS-F | TCTTCCTCAGCTGCTTCTCC |
| qdu2’,5’-OAS-R | ACTTCGATGGACTCGCTGTT |
| qduViperin-F | TCCAGTTCTGCAAGGAGGAG |
| qduViperin-R | TTCTTGAACCACCGCTCTCT |
| qduCOL11A1-F | AGAAGCTGGTAACCCTGGAC |
| qduCOL11A1-R | CACCTGGAGGTCCTTTAGCA |
| qduTHBS4-F | GGTCTGCAGCAGGAATCAAG |
| qduTHBS4-R | AGCTTCAGCTCATCCAGTGT |
| qduSYT11-F | GAGCTCTGACCCGTACATCA |
| qduSYT11-R | AGCGATCAAAGCTCAACACC |
| qduATP6V1A-F | TTACCCAGCCTACCTTGGTG |
| qduATP6V1A-R | GCCTTCCCTTTCGGGATTTC |
| qduPTX3-F | TCCCACCAGAGAAGACAACC |
| qduPTX3-R | CCATTTCTGCGATGCCTTCA |
| qduGAPDH-F | CAAGGCTGAGAATGGGAAACTT |
| qduGAPDH-R | GCATCTGCCCACTTGATGTT |
