# Supplementary material for: Duck Tembusu Virus Infection Promotes the Expression of Duck Interferon-Induced Protein 35 to Counteract RIG-I Antiviral Signaling in Duck Embryo Fibroblasts
Source: Front Immunol. 2021 Jul 15;12:711517. doi: 10.3389/fimmu.2021.711517 (PMC8320746; doi:10.3389/fimmu.2021.711517)
Supplement: Supplementary file 2 [file Table_2.docx]

**Table S2**

The sequences of siRNAs used in the study.

| siRNA | Sequences (5' to 3') | Start | Stop |
| --- | --- | --- | --- |
| siduIFI35-1  siduIFI35-2  siduIFI35-3  siNegative control | 5' GGAUACAACAGGUGAAGAATT 3'  5' UUCUUCACCUGUUGUAUCCTT 3'  5' GGGAGGAUCCGAUGAUGAUTT 3'  5' AUCAUCAUCGGAUCCUCCCTT 3'  5' GAGGAGAGGUCACUAACAUTT 3'  5' AUGUUAGUGACCUCUCCUCTT 3'  5' UUCUCCGAACGUGUCACGUTT 3'  5' ACGUGACACGUUCGGAGAATT 3' | 179  362  932 | 198  380  950 |
